# Accelerated Wound Healing with a Bilayer Biodegradable Synthetic Matrix in Post-Surgical Diabetes-Related Foot Wounds: A Randomised Controlled Trial

**DOI:** 10.3390/biomedicines14092130

**Published:** 2026-09-21

**Authors:** Frank P. Guerriero, Robyn A. Clark, Michelle Miller, Christopher L. Delaney

**Affiliations:** 1 Department of Vascular and Endovascular Surgery, Flinders Medical Centre, Flinders Drive, Bedford Park, SA 5042, Australia; christopher.delaney@flinders.edu.au; 2 College of Health and Enablement, Flinders University, GP Box 2100, Adelaide, SA 5001, Australia; robyn.clark@flinders.edu.au (R.A.C.); michelle.miller@flinders.edu.au (M.M.); 3 College of Medicine and Public Health, Flinders University, GP Box 2100, Adelaide, SA 5001, Australia

**Keywords:** limb salvage, diabetes-related foot wounds, skin substitute, wound healing matrix

## Abstract

**Objective:** Early case series using a synthetic bilayer biodegradable matrix (BTM) for diabetes-related neuropathic and neuroischemic foot wounds have shown encouraging outcomes. This study reports results from a single-centre randomised controlled trial evaluating its efficacy in a diabetes-related foot wound cohort. **Methods:** Participants with moderate-to-severe post-surgical diabetes-related foot wounds (Society of Vascular Surgery WIfI grade 3 or 4) were randomised to BTM plus usual standard of care (USOC) or USOC alone. Primary outcomes were complete wound healing and amputation rates at 12 months; secondary outcomes included time to healing, wound surface area reduction, and infection rates. (Ethics approval 2021/HRE00214, ACTRNNo. 12621001613897). **Results:** Between May 2022 and September 2024, 64 participants were enrolled (31 BTM + USOC, 30 USOC alone; three did not complete treatment). At 12 months, complete healing occurred in 64.5% of BTM-treated wounds versus 56.7% of USOC wounds (*p* = 0.53, non-significant), with no significant difference in amputation rates. Post hoc Kaplan–Meier analysis of wounds with a starting surface area greater than 10 cm^2^ showed significantly faster healing in the BTM group (median 219 days) versus USOC alone (312 days, log-rank *p* = 0.037). A multivariate Cox proportional hazards regression, adjusting for WIfI score, age and starting wound area, confirmed this effect (HR 2.67, 95% CI 1.04–6.86, *p* = 0.04), with large BTM-treated wounds healed approximately 2.7 times faster than USOC alone. **Conclusions:** In this exploratory, post hoc analysis, BTM was associated with a reduction in time to complete healing in large post-surgical diabetes-related foot wounds compared with USOC. Given the post hoc nature of this finding, confirmation in a prospectively powered trial is warranted.

## 1. Introduction

Diabetes-related foot wounds (DFWs) have been globally established as a leading cause of major amputation of the lower limb [1].

The pathophysiology underlying the development of diabetic foot wounds is complex, with multiple disease factors often contributing to an environment that hinders normal wound healing. These impaired conditions increase the risk of infection and the likelihood of progressive tissue loss [2,3,4].

In more severe forms of disease, the contributory aetiological states of peripheral arterial disease, neuropathy and glycaemic dysregulation of the local immune tissue environment predispose the foot and associated tissues to uncontrolled limb-threatening infection and/or gangrene [3,4]. Management of such circumstances frequently requires aggressive surgical removal of the infected and non-viable tissue to facilitate source control and prevent life-threatening systemic compromise [4,5]. The resulting post-surgical wounds are often extensive, and present a risk of further complications related to the risk of infection, poor wound healing, and proximal amputation of the affected limb.

With the increased risk of limb loss, placing importance on timely wound healing in the DFW cohort, there remains ongoing interest in the development of clinically effective treatments. Although evidence supporting cellular, acellular and matrix-like product (CAMP) technologies for diabetes-related foot wounds is growing, most studies focus on lower-severity chronic wounds and offer limited relevance to the post-surgical DFW cohort [6,7].

A recent entrant to the CAMP group of technologies is a bilayer biodegradable synthetic matrix (NovoSorb^®^ BTM, PolyNovo Biomaterials Pty Ltd., Port Melbourne, Australia), originally developed to support wound healing in large-surface-area burns [8]. In addition to burn treatment, NovoSorb BTM (BTM) has seen growing utility in supporting tissue regeneration in plastic and reconstructive surgery [9,10].

BTM is a biodegradable polyurethane matrix with a sealing membrane that maintains moisture and an open-cell foam that supports host tissue ingrowth. Once the matrix is fully integrated, the sealing membrane is removed to allow for epithelialisation [11,12]. Its ability to cover exposed structures such as bone and tendon makes it well-suited for post-surgical diabetes-related foot wounds where deep tissues are often exposed.

Recent experience employing BTM in the treatment of post-surgical DFWs demonstrated promising rates of wound healing and low rates of progression to major limb amputation, providing the impetus for more rigorous exploration of the clinical efficacy of this technology using a randomised control trial (RCT) methodology [7,13,14].

## 2. Materials and Methods

### 2.1. Study Design

This was a single-centre, prospective RCT conducted at a major tertiary hospital, based in southern Adelaide, South Australia. This RCT aimed to: (1) evaluate the clinical efficacy of BTM plus the usual standard of care (USOC) versus USOC alone in post-surgical DFWs of moderate-to-high severity, as defined by the Society of Vascular Surgery’s (SVS) wound, ischemia and foot infection (WIfI) system; (2) establish the safety profile of BTM in this cohort [15].

### 2.2. Inclusion and Exclusion Criteria

Adults with diabetes requiring surgical management of limb-threatening foot disease—including minor amputation, incision and drainage, wound revision, or any foot surgery generating a defect unsuitable for primary closure—were eligible. Participants were excluded if they were unable to attend scheduled reviews, were under 18, were unable to provide informed consent, required primary-closure surgery, had polyurethane hypersensitivity, were pregnant or breastfeeding, and had wounds unsuitable for BTM application due to narrow-aperture deep tissue deficits. Wound suitability was determined by the treating vascular surgeon in consultation with the trial coordinator.

### 2.3. Wound Severity Assessment

Prospective wounds were screened for severity using the WIfI classification system [15]. Only participants with a calculated 12-month amputation risk of moderate (stage 3) or high (stage 4) were enrolled. Peripheral perfusion was evaluated and optimised before consideration for trial participation. WIfI scores were calculated after surgical and/or endovascular intervention.

### 2.4. Baseline Participant Characteristics

Baseline demographics, comorbidities, HbA1c, anthropometric data, and serum inflammatory markers (white cell count, monocyte count, neutrophil count and C-reactive protein) were collected at day zero and day 28.

### 2.5. Study Intervention

Participants allocated to the BTM trial arm received a single application of BTM to their index wound, applied in an operating theatre setting following surgical debridement and secured in place with surgical staples or sutures. The BTM was further bolstered using negative-pressure wound therapy (NPWT), which was applied intraoperatively.

USOC participants received sharp bedside debridement (or theatre debridement if required) followed by immediate commencement of NPWT. The comparator control treatment for USOC participants was selected based on the recommendations outlined in the International Working Group on the Diabetic Foot (IWGDF) guidelines for management of post-surgical diabetes-related foot wounds [16,17].

Both study arms received standardised NPWT using non-biodegradable polyurethane foam (Granufoam^®^, KCI/3M, San Antonia, TX, USA) as the wound interface. For BTM-treated participants, the NPWT foam interface was applied on top of the BTM sealing membrane and directly interfaced with the wound bed tissue for USOC-treated wounds. As per consensus guidelines regarding the use of NPWT in the setting of DFWs, the NPWT was set at a treatment pressure of -75 mmHg, using the continuous treatment setting on the NPWT vacuum device (ACTI V.A.C., 3M/KCI, San Antonia, TX, USA) [18]. The duration of NPWT treatment was a total of fourteen days from the commencement of trial intervention, with dressing changes performed at days two, seven and fourteen of the initial two weeks of participation.

Following the initial fourteen days of NPWT, the wounds were treated with contemporary wound-dressing products, using a wound bed preparation approach and dressings matched to the wound characteristics as directed by the governing clinical team [19,20].

All participants were provided equal access to an accredited multidisciplinary high-risk diabetes foot team, including podiatry, infectious diseases, endocrinology, diabetes education, and vascular specialists. Trial participants were provided with a podiatry-led offloading approach, customised to individual post-surgical requirements. Wound care was overseen by a nurse practitioner, with community nursing taking over after the initial 14 days of NPWT. Multidisciplinary clinical reviews occurred on day 28 and every 28 days thereafter, with follow-up continuing for up to 365 days.

### 2.6. Study Outcomes

Complete wound healing was established as the primary outcome, defined as 100% epithelialisation of the index wound, with no drainage visible on the removed dressing at time of assessment. Participants’ wounds were assessed for qualification of complete wound healing at each of the review time points (days two, seven, 14, and 28 and every 28 days thereafter). Qualification of complete wound healing, as per the above definition, was physically validated by a minimum of two members of the multidisciplinary clinical team.

Secondary clinical outcomes included >50% wound surface area reduction (SAR) at 12 weeks post study participation, time (days) to complete wound healing, major and minor amputation rates, rates of wound infection, serum inflammatory markers, and all-cause mortality and morbidity.

Wound surface area measurements were obtained using sterile acetate tracing (Opsite Flexifix, Smith+Nephew, Yorkshire, UK). Surface area (SA) was obtained through planimetry (VISITRAK Digital, Hull, UK) [21]. SAR was calculated by comparing baseline (day 2) and week 12 measurements [(area(12 weeks)/area(baseline)) × 100/area(baseline)], carrying the last observation forward for missing data or early trial exit.

The primary and secondary study outcomes were reported at the 12-month time point, unless otherwise specified.

#### Study End Points

Study participation end points included complete wound healing of the index wound, revascularisation of the index limb, return to theatre for further debridement or minor amputation related to the index wound, or major limb amputation.

### 2.7. Randomisation

Successfully enrolled trial participants were randomly allocated to one of two treatment groups, BTM plus USOC or USOC alone, and stratified by WIfI classification (grade 3 or 4). Block randomisation (using block sizes of two, four and six) generated a total allocation list of 64. Allocation was concealed from participants, the clinical team and investigators using sequentially numbered, foil-wrapped sealed envelopes, opened only after enrolment confirmation and wound suitability assessment.

### 2.8. Ethics/Trial Governance

The study was approved by the Southern Adelaide Human Research Ethics Committee (Approval 2021/HRE00214) and was registered with the Australian New Zealand Clinical Trials Registry (ACTRN12621001613897) and conducted in accordance with Good Clinical Practice requirements.

### 2.9. Statistical Analysis

The primary analysis was undertaken on an intention-to-treat (ITT) basis, as pre-specified in the trial protocol, with those lost to follow-up before 12 months having their last observation/measure carried forward. The statistical plan, developed with a biostatistician, used paired or Wilcoxon tests for within-group changes, independent t tests or Mann–Whitney U tests for between-group differences, two-sample z tests for categorical comparisons, and Kaplan–Meier with log-rank testing for time to healing (censoring non-healed or prematurely terminated cases at 365 days) [22]. Statistical significance of *p* < 0.05 was assumed.

A post hoc exploratory subgroup analysis was subsequently performed in wounds with a starting SA greater than 10 cm^2^, based on the heterogeneous nature of participant wound sizes and clinical observation of differential healing trajectories in larger wounds. Evaluation of treatment effect independence was facilitated by multivariable Cox proportional hazards regression modelling, adjusting for WIfI score, age and starting wound SA. Proportional hazards assumption was verified graphically using log-minus-log survival plots.

Statistical Package for Social Sciences version 27 (SPSS Inc., Chicago, IL, USA) was used to perform statistical analyses. Continuous data were reported as mean (SD) or median (IQR) according to normality, and categorical data as *n* (%).

#### Sample Size

The target sample size of 64 participants was calculated using www.clincalc.com, based on the results of a previously published single-centre RCT comparing the use of a bovine collagen CAMP against a placebo wet-dressing control group in a diabetic foot wound cohort that demonstrated an increased percentage of healed wounds (86.95%) when compared with controls (52.17%) [23].

## 3. Results

**Figure 1:**
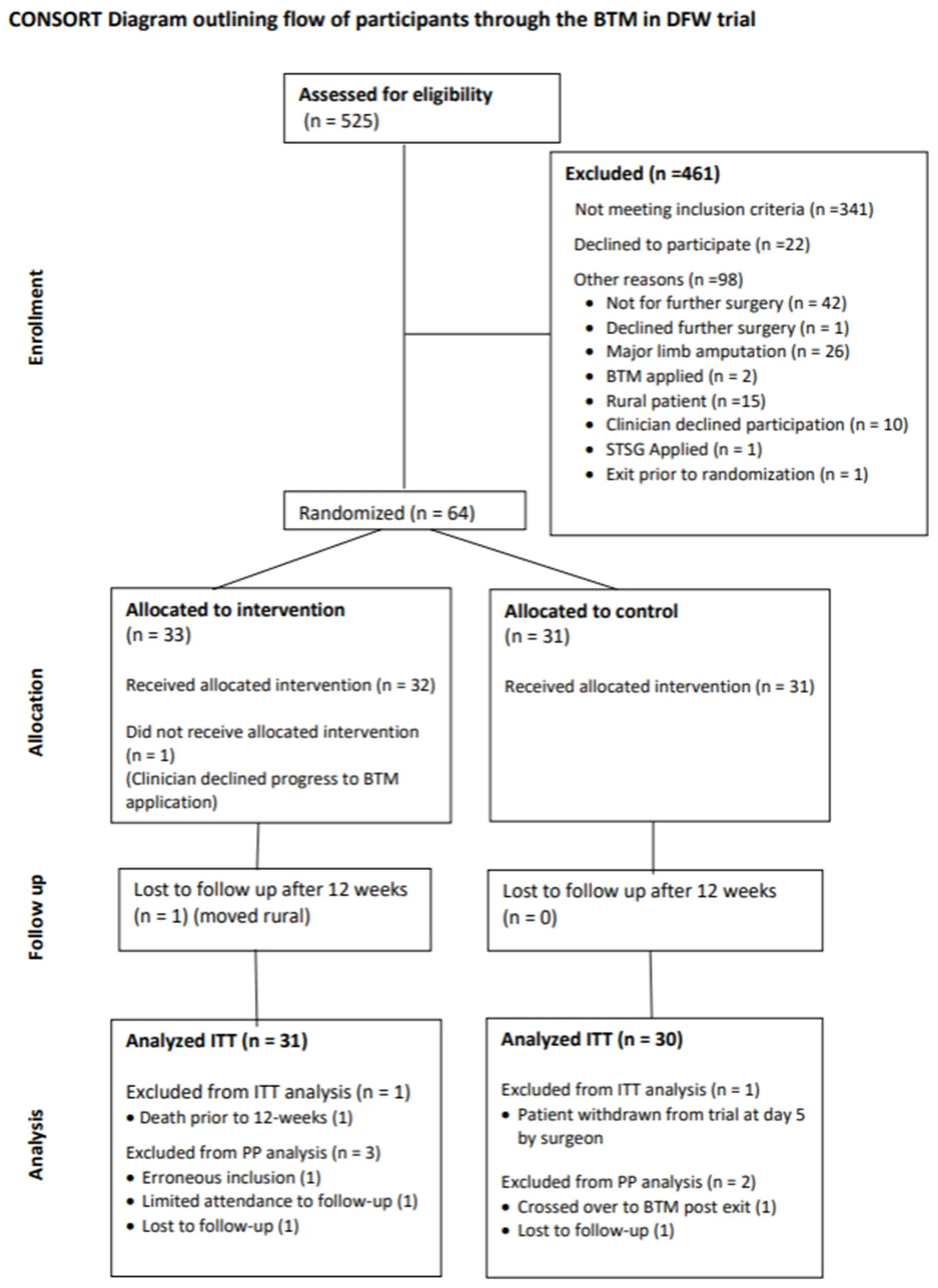
CONSORT chart.

Between May 2022 and September 2024, 525 patients were screened for eligibility for trial participation. The majority of these patients were determined to be ineligible for trial participation due to poor suitability of wound topography for BTM application (*n* = 231) and/or a WIfI grading of very low or low risk for major limb amputation. The CONSORT chart provides a summary of screening outcomes for the remaining 208 patients deemed ineligible (Figure 1).

This resulted in 86 patients eligible for enrolment, 64 of whom agreed to participate and 22 who declined participation.

### 3.1. Retention of Participants

Of the 64 participants recruited to the trial, one BTM-allocated participant did not progress to application of BTM due to concerns of ongoing clinical infection. One USOC-allocated participant was withdrawn from the trial before the day-seven outcome assessment point at the request of the governing surgeon. One participant died at day 15 post BTM application from an unrelated health complication. These participants were subsequently excluded from the ITT analysis. Exclusion of these three participants finalised the ITT analysis cohort at 61, with 31 in the BTM intervention group and 30 in the control intervention group.

### 3.2. Demographics, Comorbidities and Inflammatory Biomarkers

The study population consisted of 64 participants (mean age 40–90 years; 85.4% male). There were no statistically significant variances observed in the baseline demographics or comorbidities between the two groups. Most participants had a history of type II diabetes (95.1%), cardiac disease (98.3%) and hyperlipaemia (80.3%) (Table 1).

At day zero, the mean white cell count (WCC) was significantly higher in the USOC cohort (mean 10.8) than the BTM cohort (mean 8.5; *p* = 0.007), as were neutrophil counts (USOC 7.73 vs. BTM 5.9; *p* = 0.021). These differences resolved by day 28 (WCC *p* = 0.6; neutrophils *p* = 0.6). No significant intergroup differences were observed in baseline C-reactive protein (CRP) at day zero (*p* = 0.5) or day 28 (*p* = 0.4). The higher baseline inflammatory markers in the USOC group are likely attributable to sampling timing differences, as pre-surgical fasting requirements delayed BTM procedures by more than 24 h, placing USOC participants earlier in their infection management trajectory at the time of sampling.

### 3.3. WIfI Classification

Forty-nine of the 61 ITT trial participants were WIfI grade 4 (24 BTM, 25 USOC), representing the highest 12-month amputation risk category. The remaining 12 trial participants were WIfI grade 3 (7 BTM, 5 USOC). A breakdown of individual wound, ischemia and foot infection grading is provided in Table 2.

### 3.4. Wound Healing Outcomes

**Figure 2:**
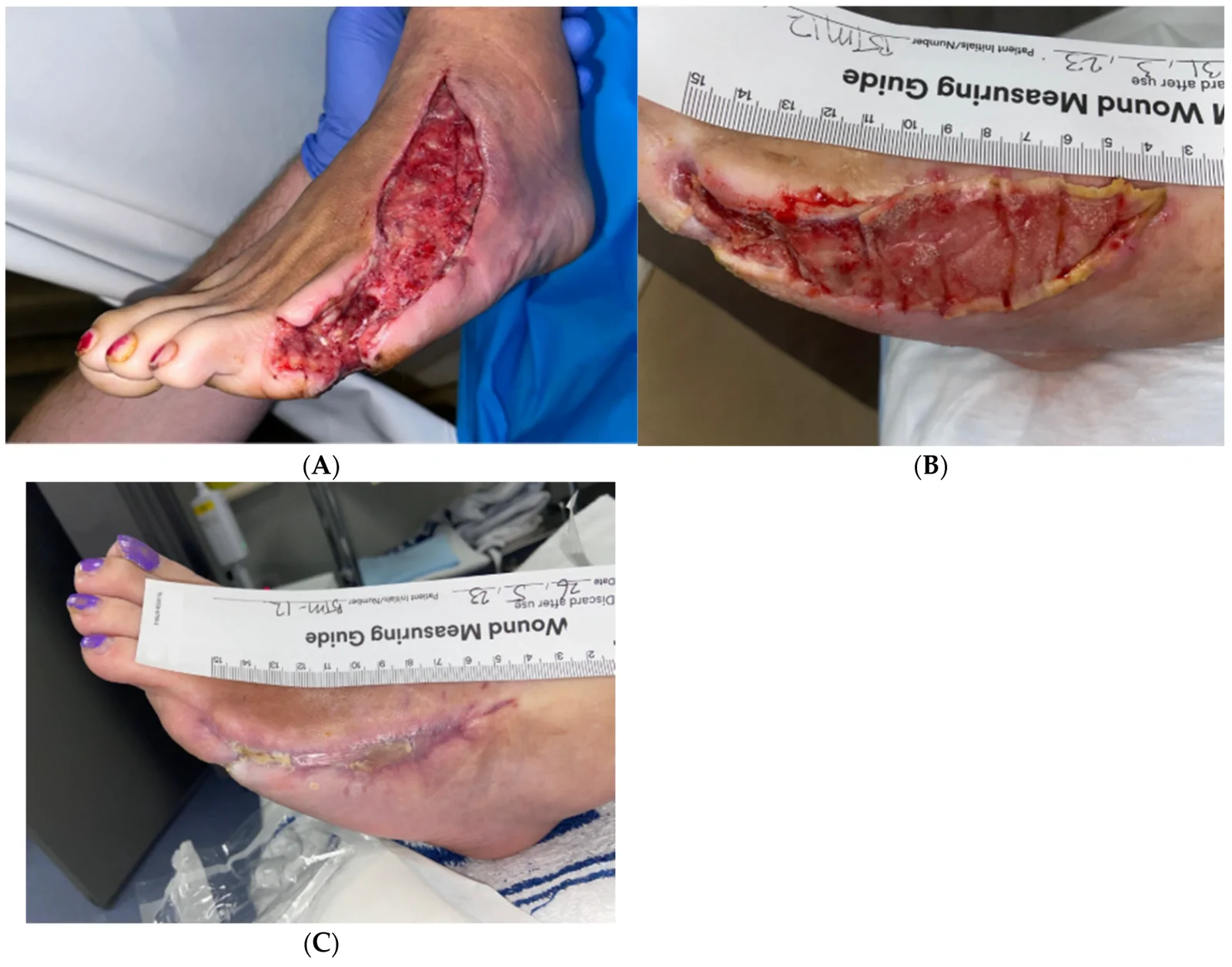
A clinical photo series of a BTM-treated foot wound at days zero (**A**) and twenty-eight (**B**) and at time of complete wound healing (day 84) (**C**). Note: the date (DD/MM/YYYY) handwritten on the rulers in images (**B**,**C**) corresponds to the date of image acquisition; the ‘Date:’ label is not visible on the ruler in image (**B**). The ruler is included for scale.

A higher proportion of BTM-treated wounds achieved complete wound healing (64.52%) compared with USOC-treated wounds (56.67%), though this difference did not reach statistical significance (*p* = 0.53) (Table 3). Subgroup analysis, stratified by starting wound SA and by WIfI severity, also failed to demonstrate a statistically significant difference in wound healing rates. All wounds that progressed to complete wound healing were healed through secondary intention, with no skin grafts or skin substitutes required (Figure 2).

### 3.5. Wound Surface Area Reduction

There was no significant difference reported in the proportion of wounds that observed a reduction in SA of ≥50% in the first 12 weeks of trial participation, in the full cohort or subgroups (Table 2).

### 3.6. Days to Complete Wound Healing

**Figure 3:**
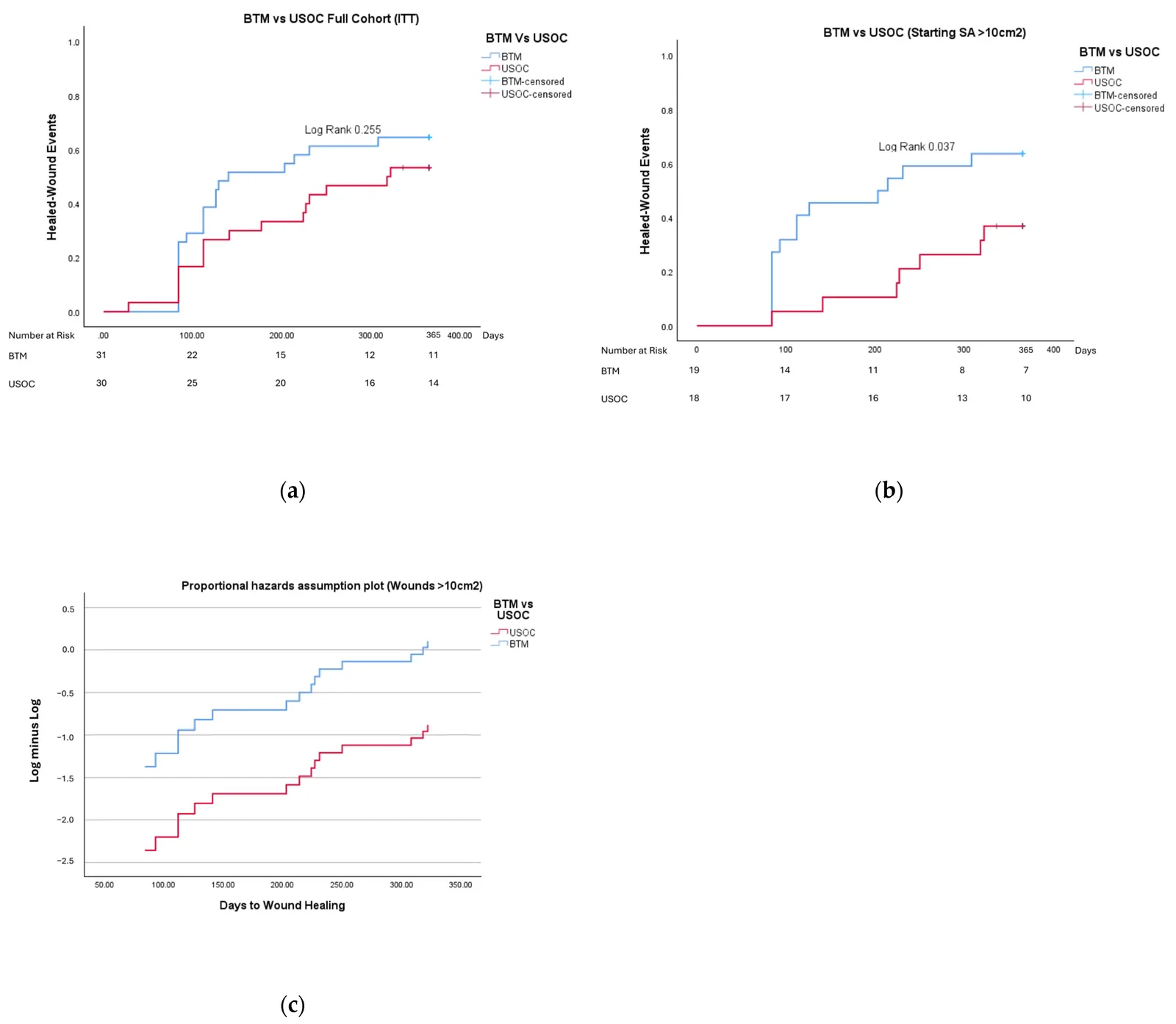
(**a**) Kaplan–Meier cumulative incidence curves for time to complete wound healing in the full intention-to-treat cohort, comparing BTM plus USOC (*n* = 30) with USOC alone (*n* = 31). Numbers at risk are shown below the *x*-axis at 100-day intervals. There was no statistically significant difference between groups (log-rank *p* = 0.255). (**b**) Kaplan–Meier analysis of time to complete wound healing in the post hoc exploratory subgroup of wounds with a starting surface area greater than 10 cm^2^ (BTM *n* = 19; USOC *n* = 18). The *y*-axis represents the cumulative proportion of healed-wound events and the *x*-axis represents follow-up time in days, censored at 365 days. BTM-treated wounds demonstrated a significantly shorter time to complete wound healing compared with USOC alone (log-rank *p* = 0.037), with a median healing time of 219 days versus 312 days, respectively. (**c**) Log-minus-log survival plot for assessment of the proportional hazards assumption in wounds with a starting surface area greater than 10 cm^2^ (BTM *n* = 19; USOC *n* = 18). The parallel trajectories of the BTM and USOC curves without crossing support proportional hazards assumptions.

There were no significant differences observed in days to complete wound healing in the full ITT cohort (Figure 3a). In the pre-specified post hoc exploratory analysis of wounds with starting SA greater than 10 cm^2^, Kaplan–Meier analysis demonstrated a significantly shorter median time to healing in BTM-treated wounds (219 days) compared with USOC alone (312 days, log-rank *p* = 0.037) (Figure 3b). Multivariable Cox proportional hazards regression, adjusting for WIfI score, age, and starting wound surface area as potential confounders, sustained and strengthened this finding (HR 2.67, 95% CI 1.045–6.865, *p* = 0.040), indicating BTM-treated wounds healed at approximately 2.7 times the rate of USOC-treated wounds, independent of these confounders. The proportional hazards assumption was confirmed by parallel log-minus-log survival curves with no crossing across the full follow-up period (Figure 3c).

### 3.7. Amputation Rates

Index wound-related major limb amputation rates were comparable between groups (BTM 9.7% *n* = 3; USOC 10%, *n* = 3; *p* = 0.96). All-cause amputation rates (BTM *n* = 6 vs. USOC *n* = 3; *p* = 0.31) and minor amputation rates (index wound-related: BTM 9.7%, USOC 13.3%; *p* = 0.64) were also not significantly different. Subgroup analysis of amputation outcomes stratified by WIfI severity failed to demonstrate any significant differences.

### 3.8. Wound Infection Rates

Index wound-related infection rates were 29% (*n* = 9) in the BTM group and 23% (*n* = 7) in the USOC group (*p* = 0.59).

### 3.9. Morbidity and Mortality

All-cause mortality was 12.9% (*n* = 4) in BTM and 13.3% (*n* = 4) in USOC (*p* = 0.963). A total of 12 post-enrolment index wound-related surgical procedures were performed in the BTM cohort, and 10 in those treated with the USOC. Post-index hospital admissions were 48 in BTM (14 index wound-related) and 44 in USOC (13 index wound-related; *p* = 0.87).

## 4. Discussion

Major limb amputation in people living with diabetes most commonly occurs in the setting of a foot wound characterised by extensive tissue damage, advancing infection, and/or peripheral arterial disease, often compounded by underlying neuropathy. This is reflected in the significantly higher amputation rates seen in patients whose combined wound, ischaemia, and infection severity places them in WIfI stages 3 or 4 [15,24,25].

While CAMP technologies offer a potentially innovative and clinically effective solution for the treatment of such severe wounds, most published clinical trials concern the treatment of chronic diabetes-related foot wounds and not the more severe acute post-surgical diabetes-related foot wounds, typically following infection source control or removal of necrotic non-viable ischemic tissue [6,26].

While the lack of significance in the primary and secondary outcomes is most likely related to an insufficiently powered cohort, the post hoc findings raise the possibility of a clinically meaningful benefit for BTM as an adjunct to USOC in large-SA post-surgical DFWs. The 93.7-day reduction in median time to complete healing suggests a therapeutic advantage in a wound environment compromised by diabetes-related immune dysfunction, impaired perfusion and biomechanical stress. The modest but directionally consistent difference in primary outcome (64.5% vs. 56.7% healed wounds) further suggests that the absence of significance reflects limited statistical power rather than an absence of true treatment effect.

The Cox regression results are important in contextualising the post hoc nature of the primary finding. The treatment hazard ratio (HR) of 2.67 was sustained and strengthened after adjustment of WIfI score, age and starting wound SA, confirming that the observed effect is independent of the baseline wound severity and participant characteristics. The consistency of the HR across unadjusted (2.50) and adjusted models (2.68, 2.67), combined with graphical confirmation of the proportional hazards assumption via log-minus-log survival, supports these findings and reduces the likelihood of a chance result.

This RCT is distinctive in that, unlike most prior CAMP studies, it predominantly enrolled severe post-surgical neuroischemic DFWs, reflected by the high proportion of WIfI grade 4 participants [6,7]. The low index wound amputation rates in both the BTM (9.7%) and USOC (10%) groups are consistent with international benchmarks, previously reported between 6% and 38% [24,27,28]. Within a cohort weighted toward the most severe forms of diabetes-related foot disease and treated under a highly effective USOC, the observed reduction in days to healing with BTM highlights the clinical value of this technology.

Further context comes from Hahn et al.’s (2021) RCT, which used a similar design and the same post-surgical DFW population [29]. Their study—the only RCT focused solely on CAMPs in this cohort—showed increased granulation tissue with CAMP plus NPWT compared with NPWT alone, with early but not sustained improvements in healing at 6 months [29]. The faster healing demonstrated in our trial builds on these early signals and strengthens the case for CAMPs in managing limb-threatening diabetes-related foot wounds.

The multifactorial nature of amputation risk in people living with diabetes, coupled with variation in facility-level care, may explain why there was no observable difference in rates of major limb amputation between treatment groups, despite the faster wound healing rates observed in the BTM-treated cohort. These findings highlight the need for larger multicentre studies to clarify BTM’s impact on limb salvage.

### 4.1. Study Limitations

A limitation of this research stems from the single-centre nature of this trial, where possible bias may exist in favour of the USOC-treated cohort, given that the trial was conducted at a tertiary referral centre with established expertise in the multidisciplinary management of diabetes-related foot complications and limb salvage. The modest sample size also stands as a further point of criticism and highlights the need for broader experience amongst other health facilities and patient populations.

A further limitation relates to the observed baseline imbalance in serum inflammatory markers between groups, with significantly higher white cell (*p* = 0.007) and neutrophil (*p* = 0.021) counts in the USOC cohort at day zero, a difference that had resolved by day 28. This disparity most likely reflects differences in the timing of blood sampling relative to surgery, with BTM-treated participants sampled over 24 h later than the USOC group due to pre-surgical fasting requirements. This delay in sampling positions BTM-treated participants later in their infection management trajectory, rather than representing a true difference in baseline inflammatory status. However, given the role of neutrophilic activity and M1 macrophage polarisation in impaired wound healing, this represents a potential confounder that limits the ability to fully attribute the observed difference in healing trajectory to BTM treatment alone [3,4].

The heterogeneous nature of the study population, facilitated by the pragmatic inclusion criteria and absence of pre-trial run-in time, is likely to have contributed to the lack of significance observed in the primary outcome. While these aspects of study design may confound the statistical power of the primary outcome, they position the findings from this research as being more relevant to a real-world clinical environment.

A technological limitation of BTM is the need to avoid folding of the sealing membrane component, which limits its applicability to deeper wound cavities, such as wounds arising from metatarsal ray resection or deeper tissue and bone debridement. This limitation is clearly reflected in the high number of post-surgical foot wound patients screened during the recruitment period and low conversion to trial participation. Subsequent release of a membrane-free version of the matrix may address this limitation and warrants further research.

Absence of a formal cost analysis serves as a key limitation. Although BTM has higher upfront product and surgical costs than the USOC, the shorter healing times and associated reductions in dressing use, nursing time, and patient travel may offset these initial expenses.

### 4.2. Implications for Clinical Practice

The results from this trial suggest that BTM may serve as a promising adjunct to USOC for the treatment of complex large-SA post-surgical DFWs and provides preliminary support for a stronger role of CAMPs in the treatment of post-surgical DFWs. BTM’s synthetic composition confers logistical advantages over biological CAMPs, avoiding biosecurity and ethical considerations associated with human- or animal-derived products. BTM typically requires only a single application, unlike many CAMPs that need repeated use, and it avoids the need for autologous harvesting or donor site wounding—a feature that may offer a supportive healing option without additional injury in a population already prone to impaired wound repair [30,31,32,33,34].

Finally, the comparable rates of post-intervention wound infections coupled with no observable difference in the intergroup rates of mortality, morbidity or post-procedural complications are reassuring and consistent with an acceptable safety profile of this technology; however, the modest sample size limits the strength of any safety conclusions that can be drawn.

## 5. Conclusions

This RCT provides preliminary evidence that BTM may be a safe and beneficial adjunct to the USOC in the treatment of moderate-to-severe post-surgical DFWs with a starting surface area greater than 10 cm^2^. While these findings are encouraging, their derision from a single centre and modest sample size situate these outcomes as hypothesis-generating rather than conclusive. Further research, incorporating larger powered multicentre studies, are needed to confirm these findings, to validate the cost efficacy of this technology compared with current standards of care, and to clarify the impact of BTM on amputation prevention in this high-risk cohort.

## Figures and Tables

**Table 1:** Baseline participant characteristics by treatment group.

Variable	BTM (*n* = 32)	%	USOC (*n* = 30)	%	*p* Value	95% CI
Demographics						
Male sex, *n* (%)	27	84.4	25	83.3	0.44	—
Age, years—mean (SD)	62.9 (17.7)		65.7 (16.5)		0.48	−10.5, 5.0
Body mass index—mean (SD)	31.6 (9.4)		30.3 (6.1)		0.53	−2.7, 5.3
Comorbidities, *n* (%)						
Type 2 diabetes	31	96.9	28	93.3	1.00	—
Type 1 diabetes	1	3.1	2	6.7	1.00	—
Chronic kidney disease	9	28.1	6	20.0	0.56	—
Insulin use	26	81.3	23	76.7	0.76	—
Prior revascularisation	10	31.3	18	55.8	0.051	−0.001, 0.49
Smoking status, *n* (%)						
Active smoker	6	18.8	7	23.3	0.93	—
Former smoker	9	28.1	6	20.0	0.81	—
Non-smoker	17	53.1	17	56.7	0.98	—
Glycaemic control						
HbA1c, % (mmol/mol)—mean (SD)	8.97 (75) (2.3)		8.51 (70) (2.2)		0.42	−0.67, 1.60
Baseline blood markers—mean (SD)						
Haemoglobin, g/L	110.9 (21.0)		113.7 (16.9)		0.56	−12.4, 6.9
C-reactive protein, mg/L	60.9 (71.3)		74.7 (90.2)		0.50	−55.4, 27.7
White cell count, ×10^9^/L	8.51 (2.68)		10.88 (3.80)		**0.007**	−4.05, −0.68
Monocyte count, ×10^9^/L	0.67 (0.29)		0.84 (0.47)		0.11	−0.36, 0.04
Neutrophil count, ×10^9^/L	5.94 (2.22)		7.73 (3.47)		0.021	−3.28, −0.28
Creatinine, µmol/L	139.1 (155.2)		98.9 (48.6)		0.17	−18.2, 98.6
Albumin, g/L	26.4 (6.5)		25.7 (5.9)		0.65	−2.4, 3.8
Day 28 inflammatory markers—mean (SD)						
C-reactive protein, mg/L	18.5 (25.5)		14.4 (14.1)		0.48	−7.7, 15.9
White cell count, ×10^9^/L	8.96 (2.90)		8.65 (2.84)		0.69	−1.3, 2.0
Neutrophil count, ×10^9^/L	5.82 (2.15)		6.10 (2.28)		0.66	−1.5, 1.0

Bold *p* values indicate statistical significance (*p* ≤ 0.05). Categorical data reported as *n* (%) and continuous data as mean (SD). Dashes indicate 95% CI not applicable for categorical comparisons using two-sample z test. BTM, synthetic bilayer biodegradable matrix; CI, confidence interval; HbA1c, glycated haemoglobin; SD, standard deviation; USOC, usual standard of care.

**Table 2:** Distribution of WIfI grade 3 and grade 4 participants by wound severity, ischemia, and infection grade sub-scores, by treatment group.

	BTM				USOC			
WIfI Grade	0	1	2	3	0	1	2	3
WIfI Grade 3								
Wound Severity	0	0	4 (33.3%)	3 (25%)	0	0	4 (33.3%)	1 (8.3%)
Ischemia	6 (50%)	1 (8.3%)	0	0	3 (25%)	2 (16.7%)	0	0
Infection Grade	1 (8.3%)	3 (25%)	3 (25%)	0	0	3 (25%)	2 (16.7%)	0
WIfI Grade 4								
Wound Severity	0	0	7 (14%)	18 (36%)	0	0	2 (4%)	23 (46%)
Ischemia	8 (16%)	4 (8%)	6 (12%)	7 (14%)	9 (18%)	8 (16%)	4 (8%)	7 (8%)
Infection Grade	3 (6%)	4 (8%)	15 (30%)	3 (6%)	2 (4%)	7 (14%)	13 (26%)	3 (6%)

WIfI, wound, ischemia, and foot infection classification. All data presented as *N* (%).

**Table 3:** Complete wound healing and wound surface area reduction outcomes at 12 months, by treatment group and WIfI severity.

Variable	BTM (*n* = 31)	BTM (%)	USOC (*n* = 30)	USOC (%)	*p* Value	95% CI
Complete wound healing at 12 months						
Complete wound healing, *n* (%)	20	64.5	17	56.6	0.53	−0.17, 0.32
WIfI stage 4 (*n* = 24 BTM; *n* = 25 USOC)						
Complete wound healing, *n* (%)	15	62.5	14	56.0	0.64	−0.21, 0.34
WIfI stage 3 (*n* = 7 BTM; *n* = 5 USOC)						
Complete wound healing, *n* (%)	5	71.4	3	60.0	0.67	−0.42, 0.65
Wound surface area reduction ≥50% at 12 weeks—ITT, *n*/*N* (%)						
Starting SA > 10 cm^2^	14/22	63.6	10/19	52.6	0.48	−0.19, 0.41
Starting SA ≤ 10 cm^2^	6/9	66.6	9/11	81.8	0.43	−0.23, 0.53
WIfI stage 4						
Starting SA > 10 cm^2^	12/19	63.2	9/18	50.0	0.42	−0.19, 0.45
Starting SA ≤ 10 cm^2^	3/5	60.0	5/7	71.4	0.68	−0.43, 0.66
WIfI stage 3						
Starting SA > 10 cm^2^	2/3	66.6	1/1	100.0	n/a ^a^	—
Starting SA ≤ 10 cm^2^	3/4	75.0	4/4	100.0	0.29	−0.21, 0.71

Categorical data reported as *n* (%) or *n*/*N* (%) where *N* denotes the subgroup denominator. Dashes indicate 95% CI not applicable. ^a^ *p* value not calculable due to insufficient participant counts in WIfI stage 3 subgroup (*n* < 5).

## Data Availability

Frank Guerriero is the guarantor of this work and, as such, had full access to all the data in the study and takes responsibility for the integrity of the data and the accuracy of the data analysis. The raw data supporting the conclusions of this article will be made available by the authors on request.

## Abbreviations

The following abbreviations are used in this manuscript:

CRP	C-reactive protein
DFW	Diabetes-related foot wound
HbA1c	Glycated haemoglobin
ITT	Intention to treat
IQR	Interquartile range
IWGDF	International Working Group on the Diabetic Foot
NPWT	Negative-pressure wound therapy
RCT	Randomised control trial
SA	Surface area
SAR	Surface area reduction
SD	Standard deviation
SVS	Society of Vascular Surgery
USOC	Usual standard of care
WIfI	Wound, ischemia and foot infection grading system
